# Socioeconomic status and prevalence of type 2 diabetes in mainland China, Hong Kong and Taiwan: a systematic review

**DOI:** 10.7189/jogh.07.011103

**Published:** 2017-06

**Authors:** Hongjiang Wu, Xiangrui Meng, Sarah H Wild, Danijela Gasevic, Caroline A Jackson

**Affiliations:** Usher Institute of Population Health Sciences and Informatics, University of Edinburgh, Edinburgh, Scotland, UK

## Abstract

**Background:**

China is estimated to have had the largest number of people with diabetes in the world in 2015, with extrapolation of existing data suggesting that this situation will continue until at least 2030. Type 2 diabetes has been reported to be more prevalent among people with low socioeconomic status (SES) in high–income countries, whereas the opposite pattern has been found in studies from low– and middle–income countries. We conducted a systematic review to describe the cross–sectional association between SES and prevalence of type 2 diabetes in Chinese in mainland China, Hong Kong and Taiwan.

**Methods:**

We conducted a systematic literature search in Medline, Embase and Global Health electronic databases for English language studies reporting prevalence or odds ratio for type 2 diabetes in a Chinese population for different SES groups measured by education, income and occupation. We appraised the quality of included studies using a modified Newcastle–Ottawa Scale. Heterogeneity of studies precluded meta–analyses, therefore we summarized study results using a narrative synthesis.

**Results:**

Thirty–three studies met the inclusion criteria and were included in the systematic review. The association between education, income and occupation and type 2 diabetes was reported by 27, 19 and 12 studies, respectively. Most, but not all, studies reported an inverse association between education and type 2 diabetes, with odds ratios (OR) and 95% confidence interval (CI) ranging from 0.39 (CI not reported) to 1.52 (95% CI 0.91 – 2.54) for the highest compared to the lowest education level. The association between income and type 2 diabetes was inconsistent between studies. Only a small number of studies identified a significant association between occupation and type 2 diabetes. Retired people and people working in white collar jobs were reported to have a higher risk of type 2 diabetes than other occupational groups even after adjusting for age.

**Conclusions:**

This first systematic review of the association between individual SES and prevalence of type 2 diabetes in China found that low education is probably associated with an increased prevalence of type 2 diabetes, while the association between income and occupation and type 2 diabetes is unclear.

The prevalence of diabetes in China has increased markedly (and much faster than in high income countries) over recent decades [1]. Nationally representative surveys indicate an increase in prevalence of diabetes in China from about 0.9% in adults aged 30 years or older in 1980 to 11.6% in adults aged 18 years or older in 2010 [2,3]. China is thought to have had the largest number of people with diabetes in the world in 2015, with extrapolation of existing data suggesting that this situation will continue until at least 2030 [4].

Socioeconomic status (SES) is a complex concept that describes the position an individual occupies in the structure of society [5]. It consists of many dimensions and is often measured by using several indicators such as income, education and occupation. SES has been recognized as an important determinant of a population’s health [6]. SES is closely linked to a wide range of health problems, including communicable and non–communicable diseases, with different strengths and directions of association in different populations [7–12]. Unlike many risk factors that have consistently shown an association with diabetes across populations, including age, overweight/obesity and physical inactivity, the association between SES and diabetes is not the same in all populations [4,13–15]. In high–income countries, type 2 diabetes is more prevalent among lower than higher socioeconomic groups [10,16–21], whereas the opposite pattern has been found in studies from low– and middle–income countries undergoing rapid economic development [22–25].

Evidence from developed countries indicates that, during the epidemiological transition, noncommunicable diseases occur initially in high SES groups, before appearing in low SES groups [26]. China has experienced extremely rapid economic development over the past 30 years and major economic inequality exists within and between regions, but it is not clear how this is associated with diabetes prevalence [27]. Previous studies have reported inconsistent associations between SES and prevalence of type 2 diabetes in China [28–31]. Understanding the association between SES and diabetes in China is necessary in order to attempt to address socioeconomic health disparities in diabetes as well as for planning approaches to primary and secondary prevention of diabetes in the Chinese population.

To our knowledge, there is no published systematic review of SES and prevalence of type 2 diabetes in China. We conducted a systematic review of cross–sectional studies to describe the association between SES (measured by education, income and occupation) and prevalence of type 2 diabetes in Chinese populations in mainland China, Hong Kong and Taiwan. Chinese people in Hong Kong and Taiwan are genetically similar to their counterparts in mainland China. However, the former are at a more advanced stage of economic development and epidemiological transition, with a larger proportion of people living in urbanised environments and developing related lifestyle habits than in China. Health care systems also differ to that of mainland China [32]. Understanding the association between SES and type 2 diabetes in Hong Kong and Taiwan is useful for helping estimate future diabetes prevalence in urban areas of mainland China.

## METHODS

### Literature search

This systematic review was conducted using the PRISMA guideline (see checklist in Appendix S1 in **Online Supplementary Document(Online Supplementary Document)**). The protocol was registered on PROSPERO and can be accessed at http://www.crd.york.ac.uk/PROSPERO/display_record.asp?ID=CRD42016047913. We carried out a systematic literature search of published studies describing the association between SES and prevalence of type 2 diabetes in mainland China, Hong Kong and Taiwan. We searched Medline (1946–May 2016), Embase (1980–May 2016) and Global Health (1973–May 2016) using a comprehensive search strategy (Appendix S2 in **Online Supplementary Document(Online Supplementary Document)**). Although the primary reviewers are Chinese, we did not include Chinese databases because other members of the research team, who provided additional review input, are not Chinese speakers. No limits were applied for language or publication time.

### Study selection and data extraction

We included cross–sectional population–based studies and baseline surveys of population–based cohort studies which: included Chinese populations in mainland China, Hong Kong or Taiwan aged 18 years or older; reported data on prevalence of type 2 diabetes or odds ratio of type 2 diabetes for populations in different SES groups; defined individual SES exposure as education, income or occupation; and were written in English. We excluded: case–control and hospital–based studies; studies limited to populations selected for specific characteristics such as hypertension or obesity; and non–English language articles. If data from the same study were reported in multiple publications, we applied the following three criteria in the order given, thereby including the publication with either: more information on the association between SES indicators and type 2 diabetes; a greater number of participants; or the most recent publication date. We did not include longitudinal studies as no longitudinal studies of incidence of diabetes in different SES groups in mainland China were identified in our pilot literature search. We conducted a pilot literature search for longitudinal studies published after 2010 based on a systematic review published in 2011, which reported no studies of incidence of diabetes and SES were identified in China [15].

Two authors (HW and XM) screened the titles, abstracts and (for potentially relevant studies) full text of articles and independently extracted key characteristics for included articles. We extracted information on: author; study year; year of publication; sample size; number of people with type 2 diabetes; demographics; participant selection; study location; SES measures; diabetes diagnosis method; outcome measures (prevalence and odds ratio); and adjustments for potential confounders. Where possible, confidence intervals for prevalence and odds ratio were calculated if they were not reported by authors. For studies reporting several models to estimate the association between SES and diabetes, the result from the model with the most complete adjustment for confounding was chosen. Disagreements were resolved by discussion between the two authors (HW and XM) with a third author acting as arbiter if a decision could not be made.

### Quality assessment

Two authors (HW and XM) independently appraised the quality of included studies using a modified Newcastle–Ottawa Scale (NOS) for cohort studies (Appendix S3 in **Online Supplementary Document(Online Supplementary Document)**) which allows a quantitative assessment of study quality [33]. This scale contains six items, categorized into three dimensions including selection, comparability, and outcome. Within the selection category, a study can be awarded one score for each of the following items: representativeness of the sample; description of the sample; and ascertainment of SES exposures. Within the comparability category, a maximum of two scores were given for the control of confounding factors. Within the outcome category, a maximum of two scores were given for the assessment of the diagnosis of diabetes and one score for the confidence intervals and probability level reported in studies. Each study was scored from 0–8, with a higher score representing higher quality.

### Synthesis of study findings

We reported type 2 diabetes prevalence and odds ratios for associations between SES indicators and type 2 diabetes for each of education, income and occupation. For education and income, we presented summary figures showing the prevalence of type 2 diabetes in the lowest and highest SES level and odds ratios of type 2 diabetes for the highest compared with lowest SES level from the model with the most complete adjustment for confounding. Ideally, we would have summarized odds ratios adjusted for age and sex only, but unfortunately few studies reported these minimally adjusted estimates, with most adjusting for additional factors. For studies reporting results only in several subgroups (eg, stratified by age and gender), we presented the result with the largest sample size. It was not possible to summarize the findings for occupation in figures, given the marked heterogeneity in definition of occupation.

For each SES indicator, the full results from each study, including stratification by urban/rural status were summarized in supplementary materials, grouped according to whether studies presented: only prevalence; only odds ratios; and both prevalence and odds ratios, and ranked from high to low quality. These figures and tables were accompanied by a narrative synthesis of the study findings, since heterogeneity between studies precluded meta–analyses.

## RESULTS

### Selection of studies

**Figure 1:**
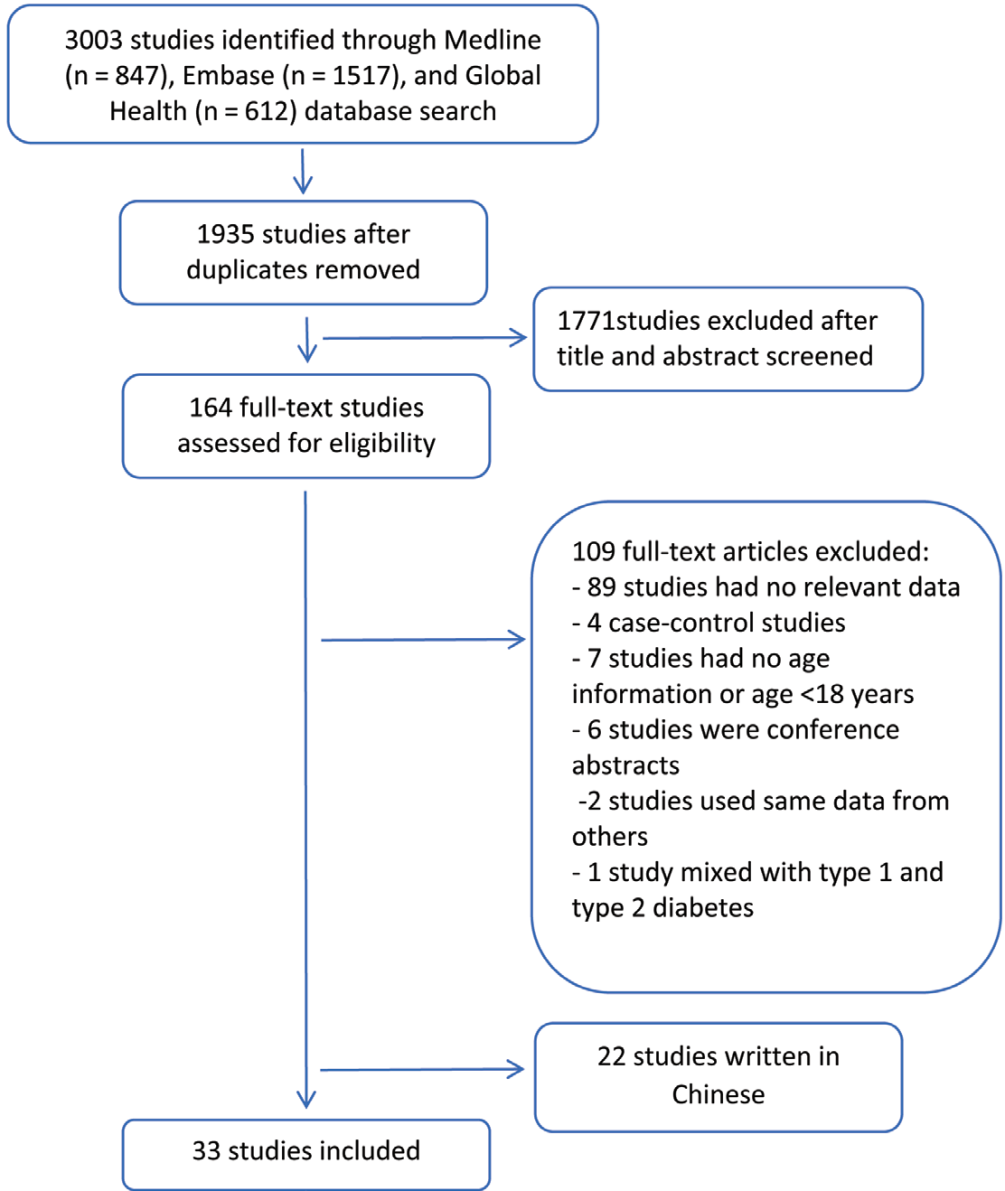
Flowchart of selection of studies in systematic review.

The literature search initially identified 3003 studies, with 1935 remaining after de–duplication. Of these, 1771 studies were excluded after title and abstract screening, and 131 further studies were excluded after full text review. Thirty–three studies met the inclusion criteria and were included in the systematic review (**Figure 1**).

### Study characteristics

An overview of the characteristics of the included studies is presented in **Table 1**. Twenty–four studies were conducted in mainland China (three in urban areas, five in rural areas and 16 in both urban and rural areas), three in Hong Kong and six in Taiwan. Study year ranged from 1986 to 2012, with a marked increase in studies on this topic over time, with 24 studies published since 2009. Sample size ranged from 988 [63] to 512 891 [41]. All studies included both men and women, but only five reported the association between SES and type 2 diabetes by sex. Self–reported diabetes and fasting blood glucose were the most commonly used methods to diagnose diabetes. Some studies used an oral glucose tolerance test (OGTT), random blood glucose and postprandial blood glucose for diagnosis of diabetes. Twelve studies provided prevalence of diabetes in different SES groups, 15 studies provided odds ratio of diabetes for different SES groups, and six provided both prevalence and odds ratios.

**Table 1:** Characteristics of studies identified in systematic review with evaluation of association between prevalence of type 2 diabetes and SES in mainland China, Hong Kong and Taiwan

Study	Study year	Participants selection	Sample, N (diabetes)	Gender (% men)	Age (mean ± SD, years)	Study location*	SES indicator	Diabetes diagnosis method	Outcome measure (prevalence/odds ratio)	Quality score
Wu et al. 2016 [34]	2007–2011	Using a random multistage stratified sampling method, 2–3 cities within each of 6 provinces from south and north China were selected, from which several communities and villages were randomly selected	23 010 (983†)	46.9	≥18 (43.0, 30.4–56.3)‡	Urban and rural	Education	Self–report or FBG	Prevalence	7
Liu et al. 2016 [35]	2001, 2010	Using a two–stage cluster random sampling method, 9 residential communities were randomly selected in Wanshoulu district in Beijing, from which all households were chosen and one person aged ≥60 was randomly selected in each household	2001: 2277 (487§), 2010: 2102 (521§)	2001: 41.4; 2010: 40.3	≥60 (67.9 ± 5.8 in 2001; 71.2 ± 6.6 in 2010)	Urban	Education	Self–report or FBG	Prevalence	5
Zhou et al. 2015 [36]	2010	Using a multistage probability sampling design, 3 communities or villages were selected in each of 4 subdistricts with probability proportional to size from each National Disease Surveillance Point; within each community 50 households were randomly selected, and one person randomly selected in each household	98 058 (12 237§)	45.7	≥18 (NR)	Urban and rural	Education, occupation	Self–report or FBG or OGTT or HbA1c	Odds ratio	6
Yu et al. 2015 [37]	2012	Using a multistage stratified random cluster sampling method, 32 counties were selected from 9 cities, from which 3 or 4 towns were randomly selected; within each town, 3 neighborhood committers were randomly selected, from each of which one village was randomly selected, before randomly selecting people aged 18 to 79 years	16 834 (1380‖)	45.9	18–79 (42.7 ± 14.5)	Urban and rural	Education, income, occupation	Self–report or FBG	Prevalence	7
Xue et al. 2015 [38]	2006, 2009	Using a stratified random cluster sampling method, people who lived in Qingdao city for at least 5 years in 3 urban areas and 3 rural areas were selected	6894 (360‡)	39.4	35–74 (51.2 ± 10.6)	Urban and rural	Education, income	HbA1c	Odds ratio	7
Xu et al. 2015 [39]	2010–2011	Using a multistage stratified random sampling method, 3 central temples and 3 counties in Chengdu region were selected from each altitude level; 4 townships were selected from each county, and within each townships 3 villages were selected, from which all people aged ≥18 were selected	1659 (106‖)	49.5	≥18 (44.0 ± 15.2)	Urban and rural	Education, income	Self–report or FBG or OGTT	Prevalence	7
Bu et al. 2015 [40]	2007–2008	Using a multistage stratified random sampling method, cities within 14 provinces in China were selected, from which 152 city districts and 112 rural villages were randomly selected; people aged ≥20 years who had lived at their current residence for ≥5 years were selected	39 071 (3254‖)	39.2	≥30 (NR)	Urban and rural	Education	Self–report or FBG or OGTT	Odds ratio	6
Bragg et al. 2014 [41]	2004–2008	People aged 30–79 were selected from five urban and five rural areas in China; these were permanent residents identified through official residential and invited by letter after extensive publicity campaigns	512 891 (30 773‖)	41.0	30–79 (NR)	Urban and rural	Education, income	Self–report or FBG or random blood glucose	Prevalence	5
Zhang et al. 2013 [28]	2005	Using a multistage stratified cluster random sampling method, 3 communities were randomly selected from two urban and one suburban district(s) in Tianjin; 3 neighborhoods were randomly selected from each community and all people who had lived in the selected neighborhoods for >5 years and were aged ≥15 years were selected	7315 (688§)	NR	20–79 (NR)	Urban	Education, income, occupation	Self–report or FBG or OGTT	Odds ratio	6
Xia et al. 2013 [42]	2010–2011	Using a stratified random sampling method, 3 communities within each of 4 districts of Haikou were randomly selected, from which 1000 people were selected	12 000 (636§)	51.0	>18 (49.1 ± 0.26¶)	Urban	Education, occupation	FBG	Prevalence	5
Wu et al. 2013 [43]	2010	Using a probability sampling design and a multistage cluster sampling method, 1 county from rural National Disease Surveillance Points (DSPs) and one district from urban DSPs were selected from 8 provinces, resulting in 64 principle sample units	13 157 (868‡)	48.1	≥50 (62.6 ± 0.3)	Urban and rural	Income	Self–report	Both	6
Wang et al. 2013 [44]	2011	Using a multistage stratified random sampling method, all townships within two counties in Yunnan province were selected and within each township 2 villages were randomly selected	4801 (341‖)	44.8	25–86 (51.1)	Rural	Education, income	Self–report or FBG or OGTT	Odds ratio	7
Cai et al. 2013 [45]	2011	Using a multistage stratified random sampling method, 1 county with high wealth and 1 county with low wealth were randomly selected in Yunnan province; people aged ≥18 were randomly selected from 20 villages within each county	9396 (614§)	46.0	≥18 (51.7 ± 19.6)	Rural	Education	Self–report or FBG	Prevalence	6
Yan et al. 2012 [46]	2009	Using a multistage random cluster sampling method, people aged ≥7 were randomly selected from 228 communities in 9 provinces	8458 (NR)	47.1	≥18 (NR)	Urban and rural	Income	FBG or HbA1c	Odds ratio	6
Chen and Chen. 2012 [47]	NR	Using a multistage random cluster sampling method, 2–12 townships were randomly selected from each 23 counties in Taiwan, within which 12–123 neighborhoods were randomly selected; within each neighborhood, 4 households were randomly selected	13 741 (NR)	57.0	18–64 (NR)	Taiwan	Occupation	Self–report	Both	6
Shi et al. 2011 [48]	2002	Using a multistage random sampling method, households were randomly selected from 6 counties and 2 cities; all people in the households were selected	2849 (79‖)	45.9	≥20 (47.0)	Urban and rural	Education	FBG	Prevalence	6
Lin et al. 2011 [49]	2004	Using a multistage random sampling method with a sampling rate proportional to size within each stage, 39 Li units were randomly selected from each 8 city districts; people were randomly selected from each sample Li	2332 (284§)	48.6	≥40 (56.9)	Taiwan	Education, income	Self–report or FBG	Prevalence	6
Kavikondala et al. 2011 [50]	2005–2008	People were randomly selected from ‘The Guangzhou Health and Happiness Association’ who are permanent residents in Guangzhou	19 818 (2193‡)	26.7	50–96 (60.4)	Urban	Education, occupation	Self–report or FBG	Odds ratio	6
Fu et al. 2011 [30]	2006–2007	All adult residents aged 18–64 were selected with exclusion of those who were temporary workers or university students not living in the county from four rural communities in Deqing, Zhejiang province	4506 (99‡)	41.4	18–64 (46.1 ± 10.0)	Rural	Education, income, occupation	Self–report or FBG	Both	7
Cai et al. 2011 [29]	2008–2010	Using a multistage stratified random sampling method, 3 counties with low, high and high level of wealth were randomly selected from Yunnan province; all townships in counties were selected and 3 villages in each township were selected by probability proportional to size, from which people aged ≥18 years were randomly selected	10 007 (657§)	46.2	≥18 (NR)	Rural	Education, income	Self–report or FBG	Odds ratio	6
Wei et al. 2010 [51]	2005	Using a multistage random cluster sampling, communities were randomly selected from 5 areas in a region in Heilongjiang	1058 (75§)	50.1	>20 (NR)	Rural	Education, income	Self–report or FBG or OGTT	Both	7
Zhou et al. 2009 [52]	2007–2008	People aged ≥20 years in 10 communities in Beijing were selected where their committees would like to cooperate with the research team	2801 (580§)	27.2	35–79 (54.7)	Urban and rural	Education, income, occupation	Self–report or FBG or OGTT	Both	7
Ning et al. 2009[53]	2001–2002, 2006	Using a stratified random cluster sampling method, people aged 35–74 years living in Qingdao city for at least 5 years were randomly selected from 3 urban districts and 4 rural counties	11 624 (1383‡)	39.6	35–74 (54.4 in 2001–2002; 51.5 in 2006)	Urban and rural	Education, income, occupation	Self–report or FBG or OGTT	Odds ratio	7
Hu et al. 2009 [54]	2000–2001	Using a multistage stratified random sampling method, 1 rural and 1 urban county within each of four provinces from North and 4 provinces from South China were randomly selected; 1 township/street was randomly selected from each county, from which people aged 35–74 years were randomly selected	15 236 (986§)	48.4	35–74 (50.1 ± 0.12¶)	Urban and rural	Education, income, occupation	Self–report or FBG	Prevalence	7
Xu et al. 2006 [31]	2000–2001	Using a multistage random sampling method, 3 urban districts and 2 rural counties were randomly selected in Nanjing, from each of which 3 streets/towns were selected; 3 villages were randomly selected in each street/town, from which people aged ≥35 y who had been a local resident for at least 5 years in each village were selected	29 340 (556§)	49.8	≥35 (NR)	Urban and rural	Education, income, occupation	Self–report	Both	6
Chou and Chi. 2005 [55]	1996	People aged ≥60 years in 6000 households were randomly selected from a continuous sample survey, which use a full list of addresses of quarters in Hong Kong as the sampling frame	2003 (246§)	47.0	≥60 (NR)	Hong Kong	Education	Self–report	Prevalence	4
Yu and Wong. 2004 [56]	NR	Households in Tai Po Hong Kong were randomly selected by telephone survey using a residential telephone directory	2670 (NR)	NR	≥20 (NR)	Hong Kong	Income	Self–report	Odds ratio	5
Woo et al. 2003 [57]	1995–1996	People aged 25–74 from 3 major of Hong Kong were randomly selected by telephone survey	988 (59§)	49.4	25–74 (45.6 ± 11.7)	Hong Kong	Education	FBG or OGTT	Prevalence	5
Chen et al. 2001 [58]	1996–1997	Using a multistage proportional stratified random cluster sampling method, people aged 50–79 years were randomly selected from 3 townships	1293 (182‡)	41.8	50–79 (63.8)	Taiwan	Education	Self–report or FBG	Odds ratio	7
Chen et al. 1999 [59]	1995–1996	Using a proportional stratified random sampling method, people aged 40–79 years were randomly selected from 6 areas	1601 (295§)	48.7	40–79 (57.4)	Taiwan	Occupation	Self–report or FBG	Odds ratio	7
Pan et al. 1997 [60]	1994	People aged ≥25 years were selected from cities and rural areas in 19 provinces	213 515 (4864§)	52.9	25–64 (NR)	Urban and rural	Income	Self–report or FBG or OGTT	Odds ratio	6
Chou et al. 1994 [61]	1991	All people aged >30 years in each village from Kin–Hu Town were selected	3236 (193§)	47.5	>30 (NR)	Taiwan	Education	FBG or OGTT	Odds ratio	5
Tai et al. 1992 [62]	1986	8 subdistricts of Ta–An District in Taipei City and 5 villages of 11 counties of Taiwan Province were randomly selected	11 478 (715§)	50.3	≥40 (NR)	Taiwan	Education, income	Self–report or FBG or postprandial blood glucose or OGTT	Odds ratio	6

SD – standard deviation, Self–report – self–reported history of type 2 diabetes or using medication for type 2 diabetes, FBG – fasting blood glucose, OGTT – oral glucose tolerance test, NR – not reported

*Urban and/or rural are in mainland China.

†Median and interquartile range.

‡Number is estimated based on the crude prevalence of diabetes.

§Number reported in the study.

‖Number is estimated based on the adjusted prevalence of diabetes.

¶Standard error.

### Quality of included studies

The quality scores of included studies ranged from 4 to 7 with a mean score of 6.0 based on the modified NOS assessment. Two studies had a highly selected study population. One selected the sample from an association for elders to represent the total older population of the study area [50], and a second study included participants who were willing to cooperate with the research team, without using any sampling techniques [52]. Fourteen studies did not report sex or age distribution, which is a limitation since both sex and age are important risk factors for type 2 diabetes [4]. Eight studies only reported crude prevalence of diabetes or unadjusted odds ratio for the association between SES and diabetes [35,42,45,48,49,55,57,61] and five studies defined diabetes solely based on self–reported diagnosis [31,43,47,55,56]. In addition, four studies did not provide confidence intervals or p values for statistical tests [35,41,42,57].

### Measures of SES

A single measure of SES was reported in 15 studies, with the remaining studies reporting data for two (10 studies) or three (8 studies) SES indicators. Education was the most commonly used indicator, being reported in 27 studies, and was classified either as highest educational level (in 21 studies) or the number of school years completed. Income was reported in 19 studies, including 15 family income measures and four personal income measures. Occupation was reported in 12 studies, but the measures of occupation differed greatly between studies, with the definition based on: job titles; skills (manual or non–manual); or a simple classification of employed and unemployed.

### Association between SES and type 2 diabetes

**Figure 2:**
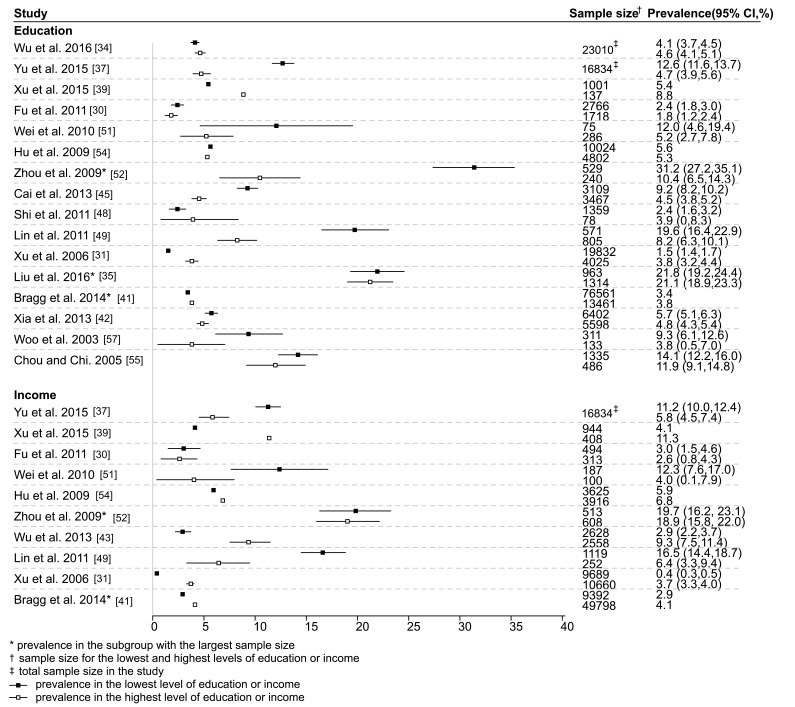
Prevalence of type 2 diabetes in the lowest and highest levels of education and income in included studies.

**Figure 3:**
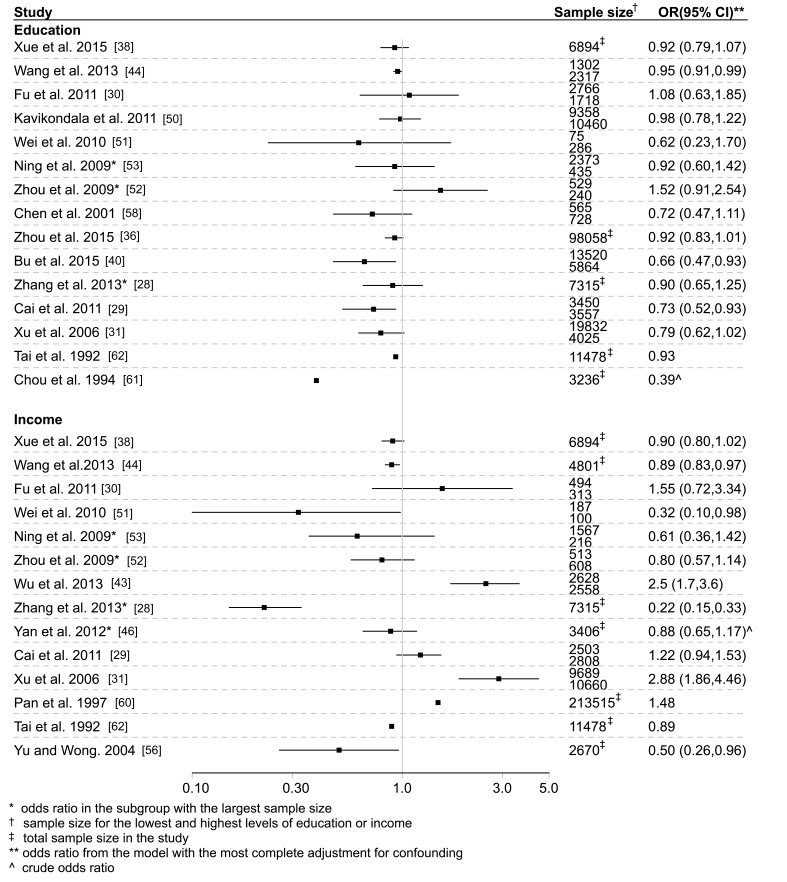
Study specific odds ratios for type 2 diabetes comparing the highest vs lowest levels of education and income in included studies.

Among the 27 studies reporting on education and type 2 diabetes, 16 reported prevalence estimates, among which five reported standardized prevalence. Fifteen studies reported odds ratios, 14 of which presented odds ratios that controlled for various potential confounders. Generally, prevalence of type 2 diabetes was higher in those with a lower compare to higher education level (**Figure 2** and Appendices S4 and S6 in **Online Supplementary Document(Online Supplementary Document))**. Most, but not all, studies reported either a significant inverse association between education level and type 2 diabetes or a possible trend toward such an association, with odds ratios (95% CI) ranged from 0.39 (CI not reported) to 1.52 (0.91, 2.54) for the highest compared to the lowest education level (**Figure 3** and Appendices S5 and S6 in **Online Supplementary Document(Online Supplementary Document)**). The studies from Hong Kong reported an inverse association [57] and no significant association [55] between education and type 2 diabetes. Among four studies from Taiwan reporting an association between education and type 2 diabetes, two reported an inverse association [49,61], and the other two reported no evidence of an association [58,62]. Among all studies, four studies reported sex–specific analyses, with two reporting that higher education was associated with increased prevalence of type 2 diabetes among men, with the opposite observed in women [34,52]. The other two studies [39,53] did not find any gender differences in the association between education and type 2 diabetes.

Of the 19 studies reporting on income and type 2 diabetes, 10 reported prevalence estimates, among which four reported a standardized prevalence. Fourteen studies reported odds ratios, all but one of which only presented odds ratios adjusted for various confounders. There was no clear pattern of prevalence of type 2 diabetes by income level across studies, with considerable inconsistency between studies (**Figure 2**, Appendices S7 and S9 in **Online Supplementary Document(Online Supplementary Document)**). Similarly, among studies reporting odds ratios, the evidence for an association between income level and type 2 diabetes was inconsistent (**Figure 3**, Appendices S8 and S9 in **Online Supplementary Document(Online Supplementary Document)**). The study from Hong Kong reported an inverse association between income and type 2 diabetes [56]. The studies from Taiwan reported an inverse association [49] and no significant association [62] between income and type 2 diabetes. Among all studies, four studies reported sex–specific analyses, among which Ning et al. [53] found a significant positive association between income and type 2 diabetes only in men in rural areas. Zhou et al. [52] and Yan et al. [46] also found a positive association in men but not in women. A fourth study did not find a gender difference, but included a very small sample size [39].

Of the 12 studies reporting on occupation and type 2 diabetes, eight reported prevalence estimates, with two reporting estimates standardized for various factors. Nine studies reported odds ratios, all of which controlled for various potential confounders. As the measures of occupation were heterogeneous, it is not easy to rank the occupation classification from high to low SES. This affects the comparability of the findings from studies reporting on occupation and type 2 diabetes and we were unable to present the results using a figure as for education and income. Zhou et al. [36] and Zhang et al. [28] found an increased risk of type 2 diabetes in retired compared to employed people after adjusting for age. Xu et al. [31] found the prevalence of type 2 diabetes was much higher in people with white collar occupations than blue collar occupations, even after controlling for confounding factors. Chen and Chen [47] found professionals had the lowest risk of type 2 diabetes compared to other kinds of occupation such as officials, salespersons and assemblers. However, most studies did not report a statistically significant association between occupation and prevalence of type 2 diabetes (Appendices S10, S11 and S12 in **Online Supplementary Document(Online Supplementary Document)**).

## DISCUSSION

This systematic review of the association between SES and prevalence of type 2 diabetes in Chinese populations in mainland China, Hong Kong and Taiwan suggests that higher education is probably associated with a decreased prevalence of type 2 diabetes. The association between income and type 2 diabetes was inconsistent between studies. While most studies found no association between occupation and diabetes prevalence, a few did report higher prevalence among people who were retired or in white–collar jobs compared to other occupations. These findings were not obviously influenced by study year or quality score.

### Explanation for findings in this systematic review

In this systematic review, most studies suggested that higher levels of education are associated with decreased prevalence of diabetes, but some found the opposite association. For example, Xu et al. [39] reported a positive association between education and prevalence of type 2 diabetes in a relatively small Tibetan population. Tibet is an undeveloped region at an earlier economic development stage compared with other parts of China, which may partly contribute to this different result. Despite being conducted in the same area and using the same methods Liu et al. [35] found a much higher prevalence of type 2 diabetes in higher education groups in people aged 60 years or older in a 2010 survey, having found no association in the 2001 survey. This study dichotomised education using a cut–off of 7 years. However, from the 1960s a large proportion of Chinese started to receive middle school education (9 years of education) [64] and so choosing 7 years as the cut–point may have different effects in different birth cohorts. Xu et al. [31] found a significantly higher crude prevalence of type 2 diabetes in people with a higher education level, but the logistic regression model revealed a non–significant inverse association after adjusting for several variables. This means that the crude positive association between prevalence of type 2 diabetes and education may have been distorted by confounding factors. Furthermore, differences in definitions of education might explain some of the heterogeneity observed between studies. It is important to note that all three studies reporting a positive association between education and prevalence of type 2 diabetes measured education as school years completed [31,35,39]. However, people may receive different economic return from school years completed compared to educational level achieved [65].

The direction of association between income and prevalence of type 2 diabetes differed between studies in our review. This is inconsistent with previous studies which has found people from high–income countries with low income were more likely to have type 2 diabetes [66,67], but an opposite association in people from low– and middle–income countries [22,68]. There are several potential explanations for the inconsistent association between income and diabetes in our review. First, unlike education, which is usually completed in young adulthood, income is unstable and sensitive to change in life circumstances and so it is not necessarily a good indicator of whole life SES [69]. Second, self–reported income is more likely to be under– or over–estimated in studies as people may consider income sensitive information and be reluctant to report it, which obviously decreases the reliability and increases the risk of non–differential bias toward a null association [70]. In addition, income is only one part of an individual’s assets and is not a very good measure among older people, especially retired people, where income is low but actual wealth can be high. Furthermore, the classification of income level is very different between studies with the lowest category ranging from <2500¥ (US$ 360) to <10 000¥ (US$ 1440) for a family’s whole year income [41,52]. Four studies in this systematic review used personal income as individual’s measure of SES [28,53,54,60]. However, total family income is believed to be more reliable than personal income, especially for young adults and women, who may not be the main earners in the family [69]. However, when applying total family income to all family members, family size should be accounted for, since for the same income, a larger family may have higher outgoing costs than a smaller family [71]. Among 15 studies reporting total family income, only one study considered family size [31]. Furthermore, China has undergone a very rapid economic development during the past several decades [72]. However, changes in an individual’s lifestyle and health–related behaviors may lag behind changes in economic conditions and may also differ in different settings.

We did not find a consistent association between occupation and prevalence of type 2 diabetes in this systematic review, though a few studies reported statistically significant findings. The classification of occupation across studies was complex and heterogeneous. Occupation in China is associated with education and income but also differing levels of physical activity that makes its classification as a risk factor for diabetes challenging.

The methods used to diagnose type 2 diabetes varied across studies, which was another source of heterogeneity between studies. Different diagnostic criteria may have a different effect on the magnitude of the association between SES and diabetes. According to the latest China nationally representative diabetes survey, around 70% of Chinese adults with diabetes were undiagnosed [3]. Thus, among the five studies that defined diabetes based on self–report, a large proportion of those with diabetes in these studies may have been erroneously assigned to the non–diabetic group and this misclassification may differ by SES groups. Bragg et al. [41] found that undiagnosed diabetes was more common among people in low education and low income groups, while the opposite was found for self–reported diabetes. People with high SES typically have more access to health resources such as routine health checks, thus they may be more likely to be aware of their health conditions. However, another study [28] did not find this difference. To more clearly examine this association, more studies reporting on the association between SES and both diagnosed and undiagnosed diabetes are required.

### Limitations of the study

Our review was limited to papers published in the English language. A systematic review of studies published in Chinese is also needed to exclude potential bias. Another important limitation is that the association between SES and diabetes was rarely the main research aim or hypothesis of most identified studies. SES was generally considered as a descriptive variable of the study sample or a potential confounder of relationships between other variables and health outcomes. It is also important to note that most prevalence estimates presented in studies were unadjusted for age, which is a key confounder of the association between SES and diabetes. Additionally, all but three studies reporting odds ratios were adjusted for various factors in addition to age and sex, many of which may lie on the causal pathway between SES and type 2 diabetes. Inclusion of these factors may have led to over–adjustment of the association between SES indicators and type 2 diabetes. A few studies in this systematic review found that the strength and direction of association between SES and prevalence of type 2 diabetes differed by sex but it is not clear whether this is consistent in different populations. A sex–specific SES gradient in health outcomes has been reported by previous studies [73]. For example, the SES gradient in prevalence of type 2 diabetes appears to be stronger in women than men in Scotland [74]. Furthermore, the scope of this review did not include the association between other indicators of individual SES (such as wealth, house condition, car and home ownership) or area–based SES measures [75].

SES indicators may have different values and implications in different urban and rural settings and in developed and undeveloped areas [76–79]. For example, people in rural areas may not need a very high education level to engage in agricultural or farming work. Also, the same level of income may have different implications for people living in developed and undeveloped areas. For example, an average monthly income of US$ 1500 provides vastly different standards of living for a family in west China compared to those living in Hong Kong. In this review, efforts were made to examine whether the association between SES and type 2 diabetes vary by study location in urban, rural mainland China, Hong Kong and Taiwan. However, the inconsistent findings and limited number of studies within each of these study geographical locations meant that no obvious patterns were observed.

### Implications for health policy and future research

Health polices for reducing socioeconomic health disparities in diabetes can only be made when the association between SES and diabetes is fully understood. This review found some evidence of an inverse relationship between education and prevalence of type 2 diabetes in Chinese populations. However, associations between income, occupation and diabetes were inconsistent. More studies, including review of those in Chinese language publications, are needed to explore the association between income and occupation and diabetes and to identify whether associations differ in different sub–groups of the population and in different regions of China. Additionally, repeated cross–sectional studies are needed to explore how associations between SES and diabetes change over time in China.

Although the association between SES and diabetes varies between countries, China is the country with the largest number of people with diabetes in the world and is undergoing rapid economic development. The epidemiological transition in China and the challenges of identifying and addressing socio–economic inequalities in health therefore have important implications for global health.

## CONCLUSIONS

This first systematic review of the association between individual SES and prevalence of type 2 diabetes in China found that low education is probably associated with an increased prevalence of type 2 diabetes. However, further work is needed to determine whether similar associations are observed with income and occupation.
