# Supplementary material for: Socioeconomic status and prevalence of type 2 diabetes in mainland China, Hong Kong and Taiwan: a systematic review
Source: J Glob Health. 2017 Jun 18;7(1):011103. doi: 10.7189/jogh.07.011103 (PMC5481892; doi:10.7189/jogh.07.011103)
Supplement: Online Supplementary Document [file jogh-07-011103-s001.pdf]

Online Supplementary Document

Wu et al. Socioeconomic status and prevalence of type 2 diabetes in mainland China, Hong Kong and Taiwan: a systematic review

J Glob Health 2017;7:011103

Appendix S1. PRISMA Checklist

Section/topic	#	Checklist item	Reported
**TITLE**			
Title	1	Identify the report as a systematic review, meta-analysis, or both.	Yes
**ABSTRACT**			
Structured summary	2	Provide a structured summary including, as applicable: background; objectives; data sources; study eligibility criteria, participants, and interventions; study appraisal and synthesis methods; results; limitations; conclusions and implications of key findings; systematic review registration number.	Yes
**INTRODUCTION**			
Rationale	3	Describe the rationale for the review in the context of what is already known.	Yes
Objectives	4	Provide an explicit statement of questions being addressed with reference to participants, interventions, comparisons, outcomes, and study design (PICOS).	Yes
**METHODS**			
Protocol and registration	5	Indicate if a review protocol exists, if and where it can be accessed (e.g., Web address), and, if available, provide registration information including registration number.	Yes

Eligibility criteria	6	Specify study characteristics (e.g., PICOS, length of follow-up) and report characteristics (e.g., years considered, language, publication status) used as criteria for eligibility, giving rationale.	Yes
Information sources	7	Describe all information sources (e.g., databases with dates of coverage, contact with study authors to identify additional studies) in the search and date last searched.	Yes
Search	8	Present full electronic search strategy for at least one database, including any limits used, such that it could be repeated.	Yes
Study selection	9	State the process for selecting studies (i.e., screening, eligibility, included in systematic review, and, if applicable, included in the meta-analysis).	Yes
Data collection process	10	Describe method of data extraction from reports (e.g., piloted forms, independently, in duplicate) and any processes for obtaining and confirming data from investigators.	Yes
Data items	11	List and define all variables for which data were sought (e.g., PICOS, funding sources) and any assumptions and simplifications made.	Yes
Risk of bias in individual studies	12	Describe methods used for assessing risk of bias of individual studies (including specification of whether this was done at the study or outcome level), and how this information is to be used in any data synthesis.	Yes
Summary measures	13	State the principal summary measures (e.g., risk ratio, difference in means).	Yes
Synthesis of results	14	Describe the methods of handling data and combining results of studies, if done, including measures of consistency (e.g., I^2) for each meta-analysis.	Yes
Risk of bias across studies	15	Specify any assessment of risk of bias that may affect the cumulative evidence (e.g., publication bias, selective reporting within studies).	No
Additional analyses	16	Describe methods of additional analyses (e.g., sensitivity or subgroup analyses, meta-regression), if done, indicating which were pre-specified.	No
**RESULTS**			
Study selection	17	Give numbers of studies screened, assessed for eligibility, and included in the review, with reasons for exclusions at each stage, ideally with a flow diagram.	Yes
Study characteristics	18	For each study, present characteristics for which data were extracted (e.g., study size, PICOS, follow-up period) and provide the citations.	Yes
Risk of bias within studies	19	Present data on risk of bias of each study and, if available, any outcome level assessment (see item 12).	Yes

Results of individual studies	20	For all outcomes considered (benefits or harms), present, for each study: (a) simple summary data for each intervention group (b) effect estimates and confidence intervals, ideally with a forest plot.	Yes
Synthesis of results	21	Present results of each meta-analysis done, including confidence intervals and measures of consistency.	Yes
Risk of bias across studies	22	Present results of any assessment of risk of bias across studies (see Item 15).	No
Additional analysis	23	Give results of additional analyses, if done (e.g., sensitivity or subgroup analyses, meta-regression [see Item 16]).	No
**DISCUSSION**			
Summary of evidence	24	Summarize the main findings including the strength of evidence for each main outcome; consider their relevance to key groups (e.g., healthcare providers, users, and policy makers).	Yes
Limitations	25	Discuss limitations at study and outcome level (e.g., risk of bias), and at review-level (e.g., incomplete retrieval of identified research, reporting bias).	Yes
Conclusions	26	Provide a general interpretation of the results in the context of other evidence, and implications for future research.	Yes
**FUNDING**			
Funding	27	Describe sources of funding for the systematic review and other support (e.g., supply of data); role of funders for the systematic review.	Yes

From: Moher D, Liberati A, Tetzlaff J, Altman DG, The PRISMA Group (2009). Preferred Reporting Items for Systematic Reviews and Meta-Analyses: The PRISMA Statement. PLoS Med 6(7): e1000097. doi:10.1371/journal.pmed1000097

For more information, visit: **www.prisma-statement.org**.

Appendix S2. Systematic review search terms

Medline (Ovid, 1946 to May Week 1 2016)

1 socioeconomic status.mp. or Social Class/

2 Socioeconomic Factors/ or Social Class/ or social position.mp.

3 Income/ or income.mp.

4 Education/ or education.mp.

5 occupation.mp. or Occupations/

6 1 or 2 or 3 or 4 or 5

7 China.mp. or China/

8 hongkong.mp or Hong Kong/

9 Taiwan.mp or Taiwan/

10 7 or 8 or 9

11 diabetes.mp. or Diabetes Mellitus, Type 2/ or Diabetes Mellitus/

12 6 and 10 and 11

Embase (1980 to 2016 Week 19)

1 socioeconomic status.mp. or social status/

2 social class.mp. or social class/

3 social status/ or social class/ or social position.mp. or socioeconomics/

4 income.mp. or income/

5 education.mp. or education/

6 occupation/ or occupation.mp.

7 1 or 2 or 3 or 4 or 5 or 6

8 China.mp. or China/

9 hongkong.mp or Hong Kong/

10 taiwan.mp. or Taiwan/

11 8 or 9 or 10

12 diabetes.mp. or diabetes mellitus/

13 7 and 11 and 12

Global Health (1973 to 2016 Week 17)

1 socioeconomic status.mp. or socioeconomic status/

2 social class.mp. or social classes/

3 social position.mp. or socioeconomic status.sh. or social classes.sh.

4 socioeconomic factors.mp. or socioeconomic status.sh. or socioeconomics.sh.

5 income/ or income.mp.

6 education/ or education.mp.

7 occupation.mp. or occupations/

8 1 or 2 or 3 or 4 or 5 or 6 or 7

9 china.mp. or China/

10 hongkong.mp or China.gl. or Hong Kong.gl.

11 Taiwan/ or Taiwan.mp.

12 9 or 10 or 11

13 diabetes.mp. or diabetes/ or diabetes mellitus/ or type 2 diabetes/

14	8 and 12 and 13

Appendix S3. Criteria list for the assessment of study quality based on modified Newcastle-Ottawa Scale for cohort studies

Selection: (Maximum 3 scores)

1) Representativeness of the sample:

a) Truly representative of the average in the target population (all subjects or random sampling or from linkage database) (1 score)

b) Somewhat representative of the average in the target population (non-random sampling)

c) Selected group of users

d) No description of the sampling strategy

2) Description of sample:

a) Study year, sample size, and age clearly described (1 score)

b) No description or not clearly described

3) Ascertainment of the exposure:

a) The ascertainment of SES exposure clearly described (1 score)

b) No description or not clearly described

Comparability: (Maximum 2 scores)

1) Confounding factors are controlled (No paper reporting both standardized prevalence and adjusted odds ratio)

a) Report both crude prevalence and adjusted odds ratio (2 scores)

b) Report standardized prevalence or adjusted odds ratio (1 score)

c) Report either of crude prevalence or crude odds ratio

Outcome: (Maximum 3 scores)

1) Assessment of the outcome:

a) Laboratory test (2 scores)

b) Record linkage (2 scores)

c) Self report (1 score)

d) No description

2) Statistical test:

a) Report confidence intervals or the probability level (p value) for prevalence or odds ratio of type 2 diabetes for population with different SES (1 score)

b) Neither confidence intervals nor the probability level (p value) are given.

Table S4: Findings of studies identified in systematic review reporting association between prevalence of type 2 diabetes and education in mainland China, Hong Kong and Taiwan

Study	Education	Adjustment	Prevalence (95% CI, %)					P-value
Wu et al. 2016 [51]	Highest level completed	2006 China population age standardized		Total	Men	Women		p<0.05[a] in all groups; p>0.05[b] in all groups
			Primary or below:	4.1 (3.7,4.5)	4.3 (3.7,4.9)	3.9 (3.4,4.4)		
			Secondary:	4.8 (4.2,5.4)	5.5 (4.6,6.4)	4.2 (3.4,5.0)		
			Matriculation or above:	4.6 (4.1,5.1)	5.8 (5.1,6.5)	3.1 (2.6,3.6)		
Yu et al. 2015 [74]	Highest level completed	6th China national population census, Jilin province population standardized (standardized for various factors)	Junior school:	12.6 (11.6,13.7)				p<0.001[a]
			Junior high school:	7.4 (6.7,8.3)				
			High school:	7.9 (7.0,8.9)				
			Undergraduate:	4.7 (3.9,5.6)				
Xu et al. 2015 [52]	School years completed	6th China national population census age standardized		Total	Men	Women		p<0.05[b] in total population and men
			0 years:	5.4	6.6	4.5		
			1-9 years:	9.2	9.1	11.9		
			≥ 10 years:	8.8	9.8	11.6		
Hu et al. 2009 [63]	Highest level completed	2000 China population census standardized (standardized for various factors)	Less than high school:	5.55				p>0.05[a]
			High school or higher:	5.28				
Cai et al. 2013 [40]	Highest level completed	Crude	Illiterate:	9.2 (8.2,10.2)				p<0.01[a], CI calculated from raw data
			Primary school:	6.1 (5.2,7.0)				
			Middle school or higher:	4.5 (3.8,5.2)				
Shi et al. 2011 [41]	Highest level completed	Crude	Primary:	2.36 (1.55,3.16)				p=0.616[a], CI calculated from raw data
			Junior school:	3.09 (2.04,4.15)				
			High school:	3.16 (1.40,4.92)				
			University:	3.90 (0,8.25)				
Lin et al. 2011 [42]	Highest level completed	Crude	Elementary school and below:	19.61 (16.35,22.87)				p<0.001[a], CI calculated from raw data
			Junior and senior high school:	11.10 (9.10,13.09)				
			College/university and above:	8.19 (6.29,10.08)				
Liu et al. 2016 [38]	School years completed	Crude		Year 2001	Year 2010			No p-value, CI calculated from raw data
			0-6 years:	21.8 (19.2,24.4)	10.9 (8.3,13.4)			
			>7 years:	21.1 (18.9,23.3)	30.1 (27.8,32.4)			
Bragg et al. 2014 [35]	Highest level completed	Age and study area structure of the study population standardized		Self-reported		Screen-detected		No p-value
				Men	Women	Men	Women	
			No formal schooling:	2.8	3.4	4.1	3.8	
			Primary school:	2.7	3.6	2.9	3.2	
			Middle school:	3.3	4.0 4.0	2.8	2.8	
			High school:	3.5	3.8	2.9	2.4	
			College or university:	4.1		2.7	2.0	
Xia et al. 2013 [39]	Highest level completed	Crude	Lower than junior high school:	5.70 (5.13,6.27)				No p-value, CI calculated from raw data
			Junior high school and above:	4.84 (4.29,5.40)				
Woo et al. 2003 [44]	Highest level completed	Crude	Primary and lower:	9.32 (6.09,12.56)				No p-value, CI calculated from raw data
			Secondary:	4.60 (2.84,6.37)				

			Post-secondary:	3.76 (0.51,7.00)	
Chou and Chi. 2005 [43]	Highest level completed	Crude	None or some elementary school:<br>Elementary graduates or above:	14.08 (12.22,15.95)<br>11.93 (9.05,14.82)	p>0.05[a], CI calculated from raw data

[a] p-value for Chi-square test.

[b] p-value for trend Chi-square test.

Table S5: Findings of studies identified in systematic review reporting odds ratio of type 2 diabetes for different education groups in mainland China, Hong Kong and Taiwan

Study	Education	Adjustment	Odds ratio (95% CI)	P-value
Xue et al. 2015 [75]	Highest level completed	Age, BMI, sex, marital status, income, alcohol consumption, smoking, cardiometabolic parameters and uric acid levels	Per level: 0.915 (0.785,1.066)	p=0.255
Wang et al. 2013 [77]	Highest level completed	Age, sex, yearly household income, ethnicity, BMI, current smoking, current drinking, hypertensive, township mean yearly income, township percent primary education or higher, township population size, township percentage of ethnic minorities	Illiteracy: 1.00<br>Primary or above: 0.95 (0.91,0.99)	p=0.027
Kavikondala et al. 2011 [36]	Highest level completed	Age, sex, study phase, WHR, BMI, maternal literacy by sex, maternal literacy by Childhood SEP and maternal literacy by late adult SEP	Junior middle or lower: 1.00<br>Senior middle or higher: 0.98 (0.78, 1.22)	No p-value
Ning et al. 2009 [53]	School years completed	Age, waist circumference, family history of diabetes, hypertension, personal income, socioeconomic status, occupation, current smoking, current drinking, leisure time physical activity, survey years and resident districts	Urban areas — Men / Women<br>≤9 years: 1.00 / 1.00<br>10-12 years: 0.97 (0.73,1.28) / 0.80 (0.62,1.03)<br>≥13 years: 0.79 (0.56,1.10) / 0.92 (0.60,1.42)<br>Rural areas — Men / Women<br>≤9 years: 1.00 / 1.00<br>10-12 years: 0.82 (0.53,1.26) / 0.92 (0.57,1.50)<br>≥13 years: 1.20 (0.66,2.17) / 0.48 (0.16,1.47)	No p-value
Chen et al. 2001 [49]	Highest level completed	Age, sex, Fukiens, Hakaas, Aborigines, family history, BMI, employment, single, smoker, drinker, regular exercise, cholesterol, triglyceride and hypertension	Illiterate: 1.00<br>Literate: 0.72 (0.47,1.11)	p>0.05
Zhou et al. 2015 [55]	Highest level completed	Region, urban/SEC cross classification, sex/age cross classification, health literacy, time since health check, minor psychiatric morbidity, weight status, number of unhealthy lifestyles, economic status, couple status and living alone	None: 1.00<br>Primary: 1.00 (0.95,1.05)<br>Secondary: 0.93 (0.87,0.99)<br>University: 0.92 (0.83,1.01)	 p>0.05<br>p<0.05<br>p>0.05
Bu et al. 2015 [76]	Highest level completed	Sex, family history of diabetes, TG per increase of 0.56mM (50mg/dl), BMI, systolic blood pressure per income of 10 mmHg, age per 10-yr increment	Elementary school: 1.52 (1.08,2.14)<br>High school: 1.42 (1.13,1.80)<br>Tertiary or above: 1.00	p<0.001<br>p<0.001

Zhang et al. 2013 [28]	School years completed	Age, sex, cigarette smoking, alcohol drinking, physical inactivity, BMI, hypertension, and family history of diabetes		Undiagnosed / Diagnosed	No p-value
			≤6 years:	0.73(0.51,1.03) / 1.11 (0.80,1.53)	
			7-9 years:	0.82 (0.59,1.16) / 0.95 (0.70,1.28)	
			> 9 years:	1.00 / 1.00	
Cai et al. 2011 [29]	Highest level completed	Age, sex, current smokers, current drinkers, hypertension, overweight, central obesity, family history of diabetes	Illiteracy:	1.00	
			Primary:	0.81 (0.65,0.96)	p<0.05
			Middle or higher:	0.73 (0.52,0.93)	p<0.05
Tai et al. 1992 [50]	Highest level completed	Age, sex, family income, exercise, physical activity, family history of diabetes and BMI	Illiterate:	1.00	
			Elementary and junior high school:	1.01	p>0.05
			Senior high school:	0.93	p>0.05
Chou et al. 1994 [45]	Highest level completed	Crude	Primary school and illiterate:	1.00	No p-value
			Junior high and above:	0.39 (0.24,0.62)	

BMI - body mass index, WHR - waist-hip ratio, SEP - socioeconomic position, SEC - socioeconomic circumstances, TG - triglyceride

Table S6: Findings of studies identified in systematic review reporting both prevalence and odds ratio of type 2 diabetes for different education groups in mainland China, Hong Kong and Taiwan

Study	Education	Outcome			P-value
Fu et al. 2011 [30]	School years completed	Crude prevalence (95% CI, %)	≤9 years:	2.4 (1.82,2.95)	No p-value, CI calculated from raw data
			> 9 years:	1.8 (1.17,2.43)	
		Odds ratio (95% CI) adjusted for age, sex, BMI, smoking alcohol drinking, and regular leisure physical activity	≤9 years:	0.93 (0.54,1.59)	p=0.787
			> 9 years:	1.00	
Wei et al. 2010 [78]	Highest level completed	Crude prevalence (95% CI, %)	Illiterate:	12.00 (4.60,19.40)	No p-value, CI calculated from raw data
			Semiliterate:	3.23 (0,9.55)	
			Primary:	6.50 (2.12,10.88)	
			Secondary:	7.73 (5.49,9.98)	
			College:	5.24 (2.66,7.83)	

		Odds ratio (95% CI) adjusted for age, gender, nationality, smoking history, alcohol consumption, marital status and family history of diabetes	Illiterate:	1.00		
			Semiliterate:	0.25 (0.03,2.08)		p=0.1989
			Primary:	0.57 (0.20,1.58)		p=0.2779
			Secondary:	0.93 (0.39,2.23)		p=0.8683
			College:	0.62 (0.23,1.70)		p=0.3521
Zhou et al. 2009 [37]	Highest level completed	Crude prevalence (95% CI, %)		Men	Women	No p-value, CI calculated from raw data
			Elementary-school/illiterate:	23.14 (15.59,30.69)	31.19 (27.24,35.14)	
			Secondary school:	22.55 (18.92,26.18)	16.15 (14.13,18.18)	
			College:	31.82 (23.84,39.79)	10.42 (6.54,14.29)	
		Odds ratio (95% CI) adjusted for age and area		Men	Women	No p-value
			Elementary-school/illiterate:	1.00	1.00	
			Secondary school:	0.67 (0.43,1.03)	1.30 (0.82, 2.06)	
			College:	0.52 (0.28,0.96)	1.52 (0.91, 2.54)	
Xu et al. 2006 [31]	School years completed	Crude prevalence (95% CI, %)	0-9 years:	1.52 (1.35,1.69)		No p-value, CI calculated from raw data
			10-12 years:	1.86 (1.50,2.22)		
			≥13 years	3.80 (3.21,4.39)		
		Odds ratio (95% CI) adjusted for age, gender, area of residence, BMI, occupation, smoking status, occupational physical activity, hospital category and health-care payment option	0-9 years:	1.00		No p-value
			10-12 years:	0.76 (0.59,0.99)		
			≥13 years:	0.79 (0.62,1.02)		

BMI - body mass index

Table S7: Findings of studies identified in systematic review reporting association between prevalence of type 2 diabetes and income in mainland China, Hong Kong and Taiwan

Study	Income	Adjustment		Prevalence (95% CI, %)				P-value
Yu et al. 2015 [74]	Family monthly income (¥)	6th China national population census, Jilin Province population standardized (standardized for various factors)	<500:	11.2 (10.0,12.4)				p<0.01[a]
			500-:	9.5 (8.4,10.7)				
			1000-:	8.1 (7.3,9.0)				
			2000-:	6.2 (5.4,7.3)				
			3000-:	5.8 (4.5, 7.4)				
Xu et al. 2015 [52]	Family yearly income (US$)	6th China national population census age standardized		Total:	Men	Women		p<0.05[b] in all groups
			<800:	4.1	5.1	2.8		
			800-1599:	5.4	5.1	5.4		
			≥1600:	11.3	14.2	8.8		
Hu et al. 2009 [63]	Personal yearly income (¥)	2000 China population census standardized (standardized for various factors)	<1,500:	5.9				p<0.01[a]
			1500-5999:	4.89				
			≥6000:	6.75				
Lin et al. 2011 [42]	Family yearly income (US$)	Crude	< 15,000:	16.53 (14.36,18.71)				p<0.001[a], CI calculated from raw data
			15,000-37,500:	8.63 (6.85,10.40)				
			> 37,500:	6.37 (3.34,9.40)				
Bragg et al. 2014 [35]	Family yearly income (¥)	Age and study area structure of the study population standardized		Self-reported		Screen-detected		No p-value
				Men	Women	Men	Women	
			< 2500:	2.4	2.9	3.7	3.9	
			2500-4999:	2.8	3.1	3.1	3.0	
			5000-9999:	2.8	3.5	2.3	2.7	
			10000-19999:	2.9	3.3	2.6	3.0	
			20000-34999:	3.1	3.4	2.7	2.8	
			≥35000:	3.8	4.1	3.4	2.7	

[a] p-value for Chi-square test.
[b] p-value for trend Chi-square test.

Table S8: Findings of studies identified in systematic review reporting odds ratio of type 2 diabetes for different income groups in mainland China, Hong Kong and Taiwan

Study	Income	Adjustment	Odds ratio (95% CI)					P-value
Xue et al. 2015 [75]	Family monthly income (¥)	Age, BMI, sex, marital status, education, alcohol consumption, smoking, cardiometabolic parameters and uric acid levels	Per level (<999,1000-2999, ≥3000):	0.896 (0.790,1.015)				p=0.085
Wang et al. 2013 [77]	Family yearly income (¥)	Age, sex, education level, ethnicity, BMI, current smoking, current drinking, hypertensive, township mean yearly income, township percent primary education or higher, township population size, township percentage of ethnic minorities	Per 1000¥:	0.89 (0.83,0.97)				p=0.01
Ning et al. 2009 [53]	Personal monthly income (¥)	Age, waist circumference, family history of diabetes, hypertension, school years completed, socioeconomic status, occupation, current smoking, current drinking, leisure time physical activity, survey and resident districts	Urban areas	Men	Women			No p-value
			≤600:	1.00	1.00			
			601-999:	1.29 (0.93,1.79)	0.81 (0.64,1.01)			
			1000-1999:	1.35 (0.93,1.95)	0.92 (0.66,1.27)			
			≥2000:	0.99 (0.61,1.59)	0.61 (0.36,1.42)			
			Rural areas	Men	Women			
			≤600:	1.00	1.00			
			601-999:	1.37 (0.88,2.12)	0.88 (0.60,1.30)			
			1000-1999:	1.29 (0.81,2.04)	1.15 (0.74,1.80)			
			≥2000:	2.00 (1.15,3.48)	1.17 (0.56,2.45)			
Zhang et al. 2013 [28]	Personal monthly income (¥)	Age, sex, cigarette smoking, alcohol drinking, physical inactivity, BMI, hypertension, and family history of diabetes		Undiagnosed	Diagnosed			No p-value
			＜1000:	4.50 (3.07,6.61)	4.56 (3.20,6.48)			
			1000-1999:	1.39 (0.95,2.04)	1.31 (0.92,1.86)			
			≥2000:	1.00	1.00			
Yan et al. 2012 [54]	Family yearly income (¥)	Crude		Men		Women		No p-value
				Aged 18-40	Aged ≥40	Aged 18-40	Aged ≥40	
			low:	1.00	1.00	1.00	1.00	
			Medium:	0.59 (0.24,1.45)	1.06 (0.77,1.46)	4.06 (0.47,34.84)	0.67 (0.49,0.93)	
			High:	0.86 (0.39,1.91)	1.51 (1.12,2.04)	2.16 (0.22,20.84)	0.88 (0.65,1.17)	
Cai et al. 2011 [29]	Family yearly income (US$)	Age, sex, current smokers, current drinkers, hypertension,	＜450:	1.00				
			450-650:	1.06 (0.81,1.46)				p>0.05
				1.22 (0.94,1.53)				p>0.05

		overweight, central obesity, family history of diabetes	＞650:		
Pan et al. 1997 [64]	Personal yearly income (¥)	Age, sex, BMI, hypertension, family history, labour, education	<2500:	1.00	
			2500-5000:	NA	
			>5000:	1.48	P=0.0001
Tai et al. 1992 [50]	Family monthly income (Taiwan$)	Age, sex, educational level, exercise, physical activity, family history of diabetes and BMI	10000:	1.00	
			10001-20000:	1.13	p<0.05
			>20000:	0.89	p>0.05
Yu and Wong. 2004 [48]	Family monthly income (HK$)	Age and sex	Low (<10,000):	2.01 (1.04,3.88)	p<0.05
			High (≥10,000):	1.00	

BMI - body mass index

Table S9: Findings of studies identified in systematic review reporting both prevalence and odds ratio of type 2 diabetes for different income groups in mainland China, Hong Kong and Taiwan

Study	Income	Outcome				P-value
Fu et al. 2011 [30]	Family income	Crude prevalence (95% CI, %)	Low:	3.0 (1.52,4.55)		No p-value, CI calculated from raw data
			Medium:	1.8 (1.37,2.24)		
			High:	2.6 (0.80,4.31)		
		Odds ratio (95% CI) adjusted for age, sex, BMI, smoking alcohol drinking, and regular leisure physical activity	Low:	1.79 (0.98,3.27)		p=0.058
			Medium:	1.00		
			High:	1.55 (0.72,3.34)		p=0.261
Wei et al. 2010 [78]	Family yearly income (¥)	Crude prevalence (95% CI, %)	<10,000:	12.30 (7.58,17.02)		No p-value, CI calculated from raw data
			10,000-20,000:	5.61 (3.47,7.74)		
			20,000-30,000:	7.08 (4.28,9.89)		
			≥30,000:	4.00 (0.14,7.86)		
		Odds ratio (95% CI) adjusted for age, gender, nationality, education, smoking history, alcohol consumption, marital status and family history of diabetes	<10,000:	1.00		
			10,000-20,000:	0.44 (0.23,0.81)		p=0.0085
			20,000-30,000:	0.60 (0.31,1.17)		p=0.1337
			≥30,000:	0.32 (0.10,0.98)		p=0.0465
Zhou et al. 2009 [37]	Family yearly income (¥)	Crude prevalence (95% CI, %)		Men	Women	No p-value, CI calculated from raw data
			<10,000:	15.51 (10.31,20.71)	19.69 (16.24,23.13)	
			10,000-30,000:	23.17 (18.51,27.84)	19.03 (16.42,21.65)	
			>30,000:	31.97 (26.10,37.83)	18.91 (15.80,22.03)	
		Odds ratio (95% CI) adjusted for age and area		Men	Women	No p-value
			<10,000:	1.00	1.00	
			10,000-30,000:	1.50 (0.92,2.45)	0.90 (0.66,1.22)	
			>30,000:	2.11 (1.25,3.57)	0.80 (0.57,1.14)	

Wu et al. 2013 [46]	Family income quintile	Crude prevalence (95% CI, %)	Lowest:	2.9 (2.2,3.7)	No p-value
			Second:	5.2 (4.2,6.3)	
			Middle:	7.1 (5.7,8.7)	
			Fourth:	7.3 (6.0,8.8)	
			Highest:	9.3 (7.5,11.4)	
		Odds ratio (95% CI) adjusted for age, sex and urban/rural residence	Lowest:	1.00	
			Second:	1.9 (1.4,2.7)	p<0.05
			Middle:	2.3 (1.7,3.3)	p<0.05
			Fourth:	2.4 (1.6,3.6)	p<0.05
			Highest:	2.5 (1.7,3.6)	P<0.05
Xu et al. 2006 [31]	Family per capita monthly income	Crude prevalence (95% CI, %)	Lower:	0.41 (0.29,0.54)	No p-value, CI calculated from raw data
			Middle:	1.39 (1.15,1.63)	
			Higher:	3.67 (3.31,4.02)	
		Odds ratio (95% CI) adjusted for age, gender, area of residence, BMI, education, occupation, smoking status, occupational physical activity, hospital category and health-care payment option	Lower:	1.00	No p-value
			Middle:	1.95 (1.27,2.99)	
			Higher:	2.88 (1.86,4.46)	

BMI - body mass index

Table S10: Findings of studies identified in systematic review reporting association between prevalence of type 2 diabetes and occupation in mainland China, Hong Kong and Taiwan

Study	Adjustment	Prevalence (95% CI, %)		P-value
Yu et al. 2015 [74]	6th China national population census, Jilin Province population standardized (standardized for various factors)	Manual labour:	7.1 (6.5,7.7)	p<0.001[a]
		Mental labour:	5.9 (5.1,6.9)	
		Other:	13 (11.8,14.2)	
Hu et al. 2009 [63]	2000 China population census standardized (standardized for various factors)	Professional:	5.95	p>0.05[a]
		Labourer:	5.4	
		Other:	5.68	
Xia et al. 2013 [39]	Crude	Mental:	4.91 (4.26,5.57)	No p-value, CI calculated from raw data
		Non-mental:	5.51 (5.00,6.01)	

[a] p-value for Chi-square test.

Table S11: Findings of studies identified in systematic review reporting odds ratio of type 2 diabetes for different occupation groups in mainland China, Hong Kong and Taiwan

Study	Adjustment	Odds ratio (95% CI)			P-value
Kavikondala et al. 2011 [36]	Age, sex, study phase, WHR, BMI, maternal literacy by sex, maternal literacy by Childhood SEP and maternal literacy by early adult SEP	Low (agricultural worker, factory work or sales and service):	1.00		No p-value
		High (administrator/manager, professional/technical, military/police):	0.99 (0.75, 1.29)		
Ning et al. 2009 [53]	Age, waist circumference, family history of diabetes, hypertension, personal income, School years completed, socioeconomic status, current smoking, current drinking, leisure time physical activity, survey and resident districts	Urban areas	Men	Women	No p-value
		Professional:	1.00	1.00	
		Not work outside the home:	1.07 (0.73,1.57)	1.48 (0.97,2.27)	
		Worker/farmer:	0.90 (0.61,1.31)	1.21 (0.77,1.91)	
		Rural areas	Men	Women	
		Professional:	1.00	1.00	
		Not work outside the home:	0.73 (0.39,1.37)	0.66 (0.26,1.68)	
		Worker/farmer:	0.65 (0.38,1.10)	0.72 (0.29,1.81)	
Chen et al. 1999 [79]	Age, sex	Unemployed:	1.30 (0.92,1.82)		p>0.05
		Employed:	1.00		
Zhou et al. 2015 [55]	Region, urban SEC cross classification, sex/age cross classification, health literacy, time since health check, minor psychiatric morbidity, weight, number of unhealthy lifestyles, highest education qualification, economic status, couple status and living alone	Employed:	1.00		
		Housewife/husband:	1.17 (1.08,1.28)		p<0.05
		Retired:	1.34 (1.24,1.45)		p<0.05
		Unemployed/student/other:	1.17 (1.10,1.24)		p<0.05
Zhang et al. 2013 [28]	Age, sex, cigarette smoking, alcohol drinking, physical inactivity, BMI, hypertension, and family history of diabetes		Undiagnosed	Diagnosed	No p-value
		Retirement:	2.01 (1.40,2.89)	3.02 (2.12,4.22)	
		Unemployment:	0.83 (0.55,1.25)	1.25 (0.84,1.78)	
		Non-manual work:	1.05 (0.70,1.55)	1.23 (0.82,1.80)	
		Manual work:	1.00	1.00	

WHR - waist-hip ratio, BMI - body mass index, SEP - socioeconomic position, SEC - socioeconomic circumstances

Table S12: Findings of studies identified in systematic review reporting both prevalence and odds ratio of type 2 diabetes for different occupation groups in mainland China, Hong Kong and Taiwan

Study	Outcome				P-value
Fu et al. 2011 [30]	Crude prevalence (95% CI, %)	Farmer:	2.3 (1.77,2.80)		No p-value, CI calculated from raw data
		Not Farmer:	1.9 (1.15,2.65)		
	Odds ratio (95% CI) adjusted for age, sex, BMI, smoking alcohol drinking, and regular leisure physical activity	Farmer:	1.10 (0.66,1.84)		p=0.723
		Not Farmer:	1.00		
Zhou et al. 2009 [37]	Crude prevalence (95% CI, %)		Men	Women	No p-value, CI calculated from raw data
		Worker/farmer:	21.20 (17.35,25.05)	19.72 (17.36,22.09)	
		Other:	27.60 (21.26,33.94)	20.42 (17.47,23.36)	
		Office working:	29.20 (21.56,36.84)	14.47 (9.90,19.05)	
	Odds ratio (95% CI) adjusted for age and area		Men	Women	No p-value
		Worker/farmer:	1.00	1.00	
		Other:	1.21 (0.81,1.82)	1.03 (0.80,1.33)	
		Office working:	1.35 (0.85,2.12)	0.85 (0.56,1.31)	
Chen and Chen. 2012 [47]	Crude prevalence (%)	Professionals:	1.0		No p-value
		Senior officials and managers:	3.7		
		Clerks:	1.3		
		Salespersons, demonstrators and models:	3.3		
		Craft and related workers:	2.3		
		Plant and machine operators and assemblers:	3.5		
		Personal and protective services workers:	1.7		
		Elementary occupations:	3.6		
		Skilled agricultural and fishery workers:	3.9		
	Odds ratio (95% CI) adjusted for age, sex, cigarette use, and alcohol use	Professionals:	1.00		
		Senior officials and managers:	1.79 (1.05,3.06)		$p<0.05$
		Clerks:	1.48 (0.83,2.65)		$p>0.05$
		Salespersons, demonstrators and models:	2.72 (1.45,5.11)		$p<0.01$
		Craft and related workers:	1.70 (0.94,3.07)		$p>0.05$
		Plant and machine operators and assemblers:	2.47 (1.38,4.39)		$p<0.01$
		Personal and protective services workers:	1.38 (0.71,2.69)		$p>0.05$
		Elementary occupations:	1.80 (0.89,3.63)		$p>0.05$
		Skilled agricultural and fishery workers:	1.14 (0.60,2.17)		$p>0.05$
Xu et al. 2006 [31]	Crude prevalence (95% CI, %)	Blue Collar:	0.64 (0.51,0.76)		No p-value, CI calculated from raw data
		Service:	1.28 (0.92,1.64)		
		White Collar:	4.08 (3.70,4.47)		
	Odds ratio (95% CI) adjusted for age, gender, area of residence, BMI, education, smoking status, occupational physical activity, hospital category and health-care payment option	Blue Collar:	1.00		No p-value
		Service:	1.47 (0.99,2.18)		
		White Collar:	1.39 (1.05,1.85)		

BMI - body mass index
